# Wastewater surveillance beyond COVID-19: a ranking system for communicable disease testing in the tri-county Detroit area, Michigan, USA

**DOI:** 10.3389/fpubh.2023.1178515

**Published:** 2023-06-02

**Authors:** Zachary Gentry, Liang Zhao, Russell A. Faust, Randy E. David, John Norton, Irene Xagoraraki

**Affiliations:** ^1^Department of Civil and Environmental Engineering, Michigan State University, East Lansing, MI, United States; ^2^Oakland County Health Division, Pontiac, MI, United States; ^3^Wayne State University School of Medicine, Detroit, MI, United States; ^4^Great Lakes Water Authority, Detroit, MI, United States

**Keywords:** wastewater surveillance, communicable disease (CD), ranking system, COVID-19, emerging disease

## Abstract

**Introduction:**

Throughout the coronavirus disease 2019 (COVID-19) pandemic, wastewater surveillance has been utilized to monitor the disease in the United States through routine national, statewide, and regional monitoring projects. A significant canon of evidence was produced showing that wastewater surveillance is a credible and effective tool for disease monitoring. Hence, the application of wastewater surveillance can extend beyond monitoring SARS-CoV-2 to encompass a diverse range of emerging diseases. This article proposed a ranking system for prioritizing reportable communicable diseases (CDs) in the Tri-County Detroit Area (TCDA), Michigan, for future wastewater surveillance applications at the Great Lakes Water Authority's Water Reclamation Plant (GLWA's WRP).

**Methods:**

The comprehensive CD wastewater surveillance ranking system (CDWSRank) was developed based on 6 binary and 6 quantitative parameters. The final ranking scores of CDs were computed by summing the multiplication products of weighting factors for each parameter, and then were sorted based on decreasing priority. Disease incidence data from 2014 to 2021 were collected for the TCDA. Disease incidence trends in the TCDA were endowed with higher weights, prioritizing the TCDA over the state of Michigan.

**Results:**

Disparities in incidences of CDs were identified between the TCDA and state of Michigan, indicating epidemiological differences. Among 96 ranked CDs, some top ranked CDs did not present relatively high incidences but were prioritized, suggesting that such CDs require significant attention by wastewater surveillance practitioners, despite their relatively low incidences in the geographic area of interest. Appropriate wastewater sample concentration methods are summarized for the application of wastewater surveillance as per viral, bacterial, parasitic, and fungal pathogens.

**Discussion:**

The CDWSRank system is one of the first of its kind to provide an empirical approach to prioritize CDs for wastewater surveillance, specifically in geographies served by centralized wastewater collection in the area of interest. The CDWSRank system provides a methodological tool and critical information that can help public health officials and policymakers allocate resources. It can be used to prioritize disease surveillance efforts and ensure that public health interventions are targeted at the most potentially urgent threats. The CDWSRank system can be easily adopted to geographical locations beyond the TCDA.

## 1. Introduction

Since the beginning of the coronavirus disease 2019 (COVID-19) pandemic, wastewater surveillance has been consistently applied to monitor severe acute respiratory syndrome coronavirus 2 (SARS-CoV-2) viral RNA worldwide (1–10). Wastewater surveillance epidemiology is a translation of the theory that human wastewater can serve as a representative community-composite sample to monitor fluctuations of disease incidence. A pathogen that can be detected in bodily fluids, including excreta, urine, sputum, and saliva, has the potential to be detected and thus, monitored (2, 11–14). Wastewater surveillance and epidemiology has a diverse range of benefits, including (1) circumventing the need for mass clinical testing, (2) conserving health, economic, and societal resources, (3) providing unbiased and unspecific monitoring of disease incidence regardless of symptomatic or asymptomatic conditions, and (4) providing early warnings of impending disease surges (4, 5, 7, 10, 12, 15). Wastewater surveillance has been extraordinarily successful at monitoring multiple pathogens, including SARS-CoV-2 (2, 4–7, 11, 16), hepatitis A and hepatitis E (17), herpesviruses (18), poliovirus (19, 20), and others. Despite its great potential, most wastewater disease monitoring to date has been limited to SARS-CoV-2. Notably, recent exceptions encompass poliovirus (21) and monkeypox virus (22–24). Thus, it is paramount that the adoption and integration of this scientifically-validated methodology is accelerated, particularly among emerging disease, neglected disease, or diseases of high outbreak potential.

Communicable diseases (CDs), for instance, tuberculosis (TB) and sexually transmitted infections (STIs), are among the leading causes of death and disability worldwide, according to the WHO (who.int). CDs are caused by microorganisms including bacteria, viruses, fungi, or various parasites that can be transmitted widely and quickly within human populations (25). Some infectious diseases are transmitted through “bites” from insect vectors, while others can be caused by ingesting contaminated food or water (who.int). The WHO, U.S. NIH, U.S. AID, U.S. CDC, and the international scientific community has long recognized the need to develop a comprehensive education, prediction, and prevention system for CDs (13, 26, 27).

A few studies have developed methodologies for ranking CD threats to the public (28–30). However, these systems have limitations and cannot be directly used by local health department to make decisions regarding appropriate targets for wastewater surveillance. Briefly, they relied heavily on subjective assessments of weights given by experts to multiple parameters. They were lacking critical quantitative information such as incidence of diseases based on clinical data, and basic reproduction numbers of CDs. Besides, most parameters were assigned a value according to the Delphi Method, which consists of gathering expert opinions to weight a disease on a parameter then multiplied by a scale of numbers such as 1–5 (29) or 0–7 (31) in terms of level of importance.

**Figure 1:**
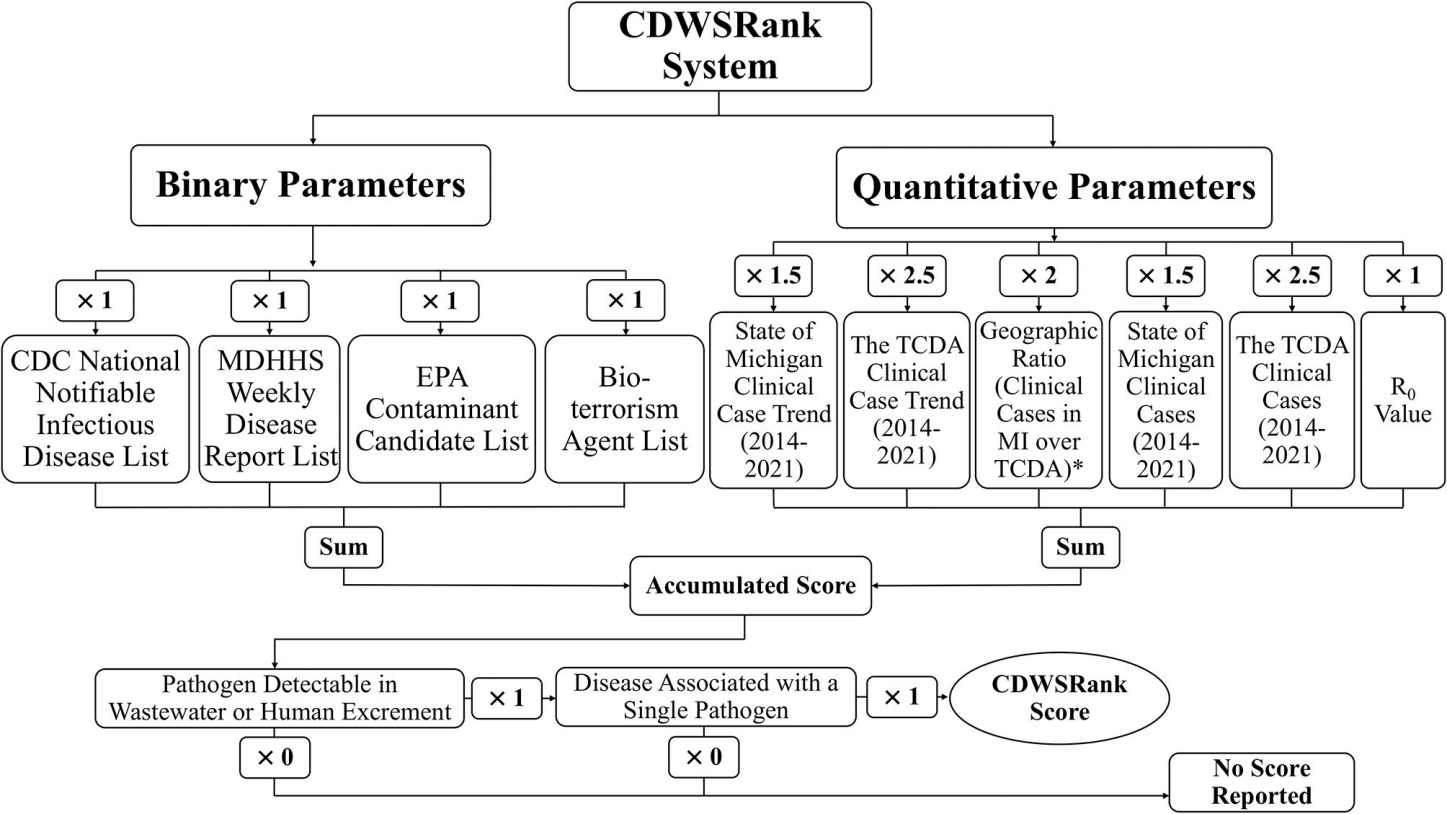
Overall schematic of CDWSRank system. *marked parameter indicates that the annual caseload for Michigan from 2014-2021 was divided by the annual caseload for the TCDA, where the average of those was then taken for downstream analyses. The A+1 value was assigned to those CDs with an average ratio of <1.

The objective of this study is to develop a comprehensive communicable disease ranking system (“CDWSRank” system) that prioritizes CDs for wastewater surveillance (Figure 1). To this end, we investigated 96 CDs in the Tri-County Detroit Area (TCDA), Michigan, Unites States, reported through the Michigan Disease Surveillance System (MDSS). All CDs were ranked through the CDWSRank system, which involved 2 categories of parameter: binary and quantitative. Binary parameters examine the presence or absence of CDs in the following inventories: (1) CDC National Notifiable Infectious Disease and Conditions List (NNIDCL), (2) Michigan Department of Health and Human Services (MDHHS) Weekly Disease Report, (3) EPA Contaminant Candidate List (CCL), (4) CDC bioterrorism agents list, (5) pathogen's detectability in wastewater or excreta, and (6) association of disease with single or multiple pathogens. Quantitative parameters include: (1) clinical case trend in Michigan, (2) clinical case trend in the TCDA, (3) ratio of clinical case incidence between Michigan and the TCDA (geographic ratio), (4) annual clinical cases in Michigan, (5) annual clinical cases in the TCDA, and (6) the R_0_ (basic reproduction number) of the disease.

The CDWSRank system is one of the first of its kind to provide an empirical method for selecting CDs for wastewater surveillance, in geographies serviced by centralized wastewater collection and treatment. To demonstrate the importance of site-specific ranking, CD trends were analyzed for both the TCDA and Michigan as a whole for the period between 2014 and 2021. This manuscript will moreover summarize wastewater sampling methods based on pathogen type. Ultimately, this article should contribute to the reduced impact of CDs by procuring valuable information for public health practitioners, researchers, and medical professionals.

## 2. Materials and methods

### 2.1. Communicable disease data acquisition

**Figure 2:**
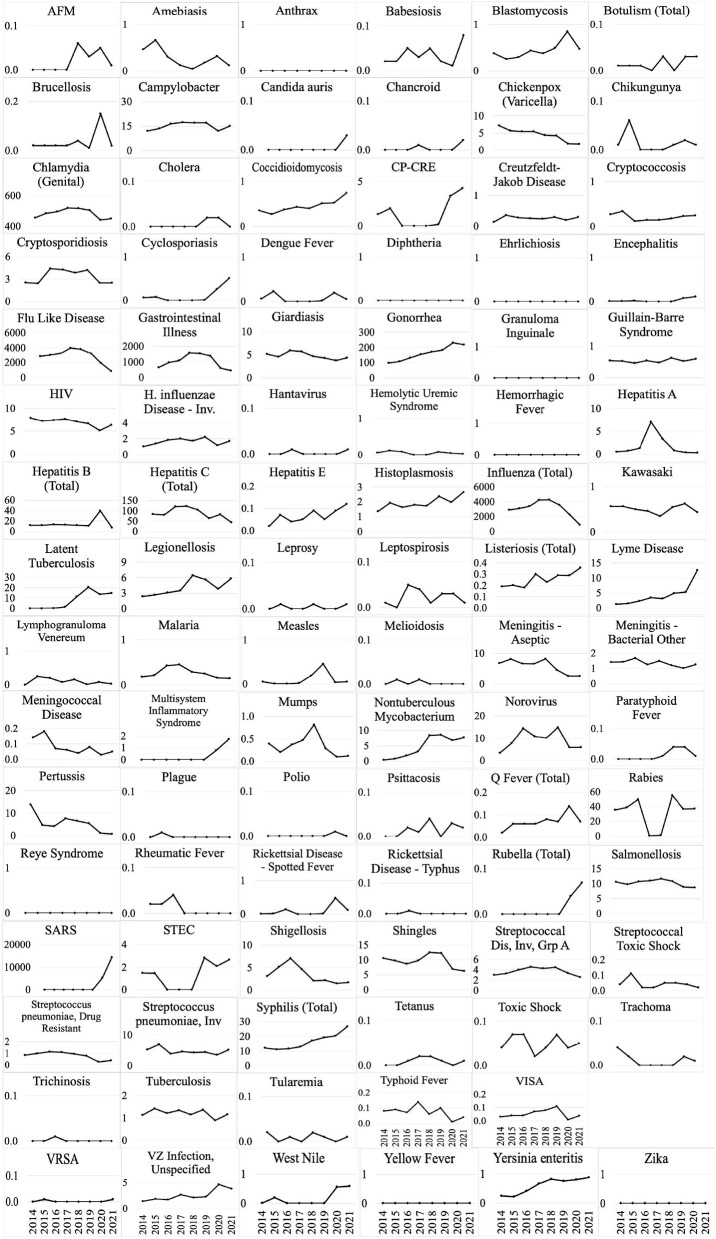
Disease incidence (per 100,000) for 95 CDs between 2014 and 2021 in the state of Michigan (Disease incidence for Monkeypox was unavailable during this period).

**Figure 3:**
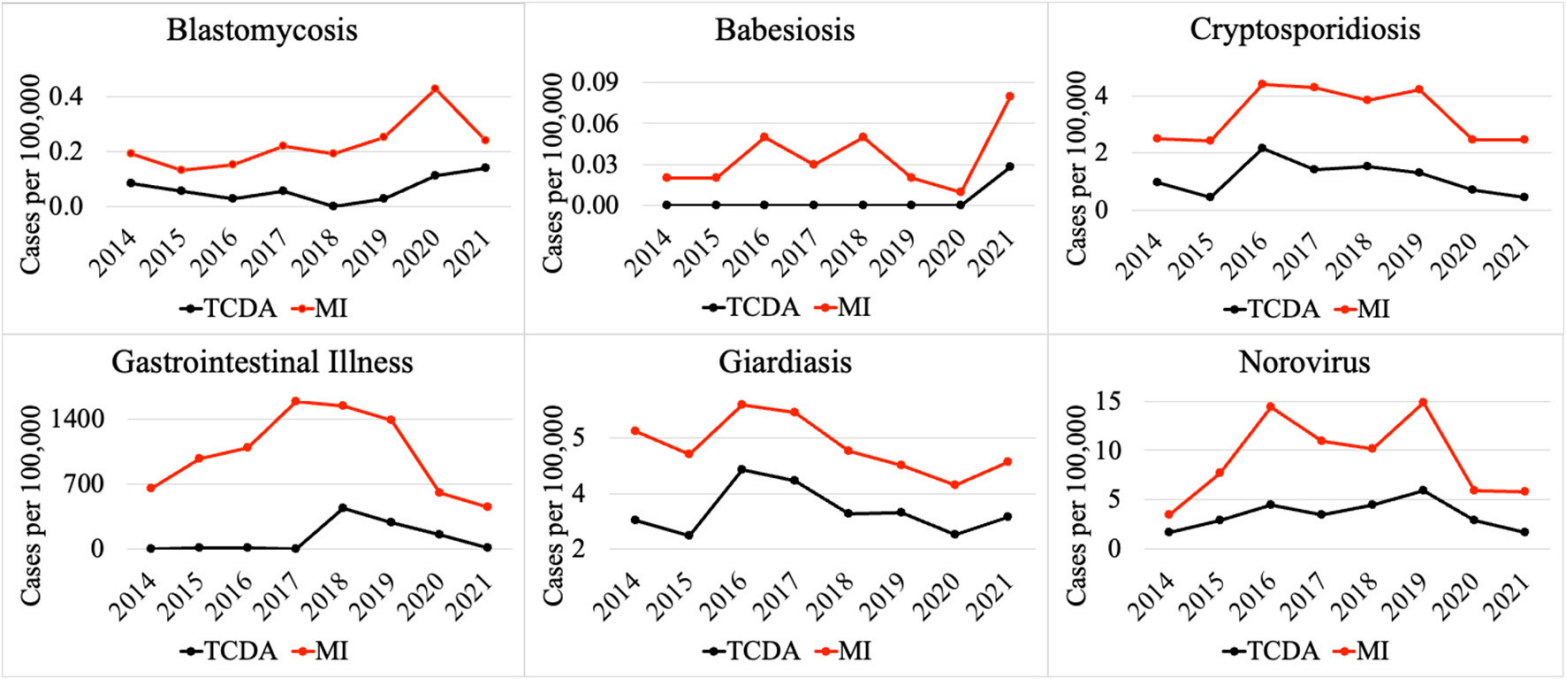
Comparison of selected CDs incidences (per 100,000) between TCDA and MI (ratio < 1).

**Figure 4:**
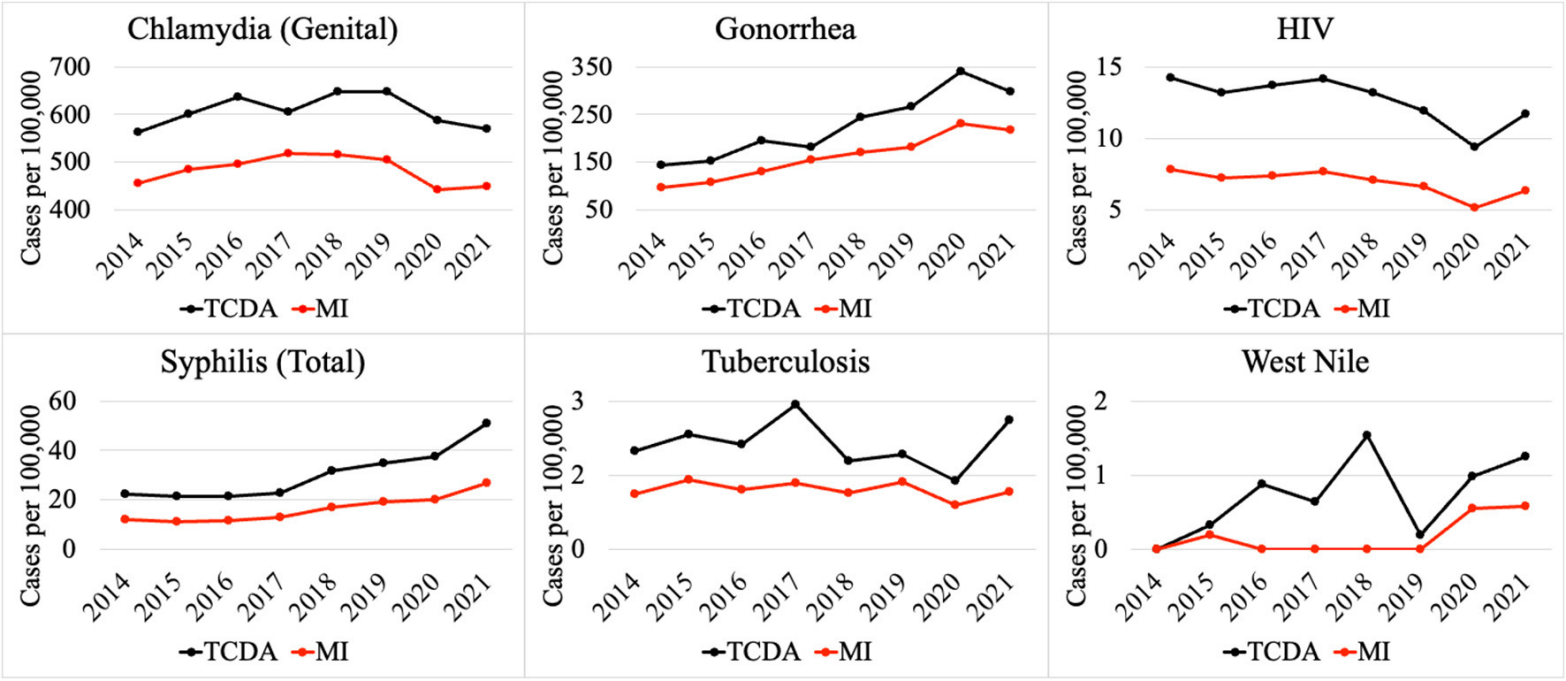
Comparison of selected CDs incidences (per 100,000) between TCDA and MI (ratio > 1).

**Figure 5:**
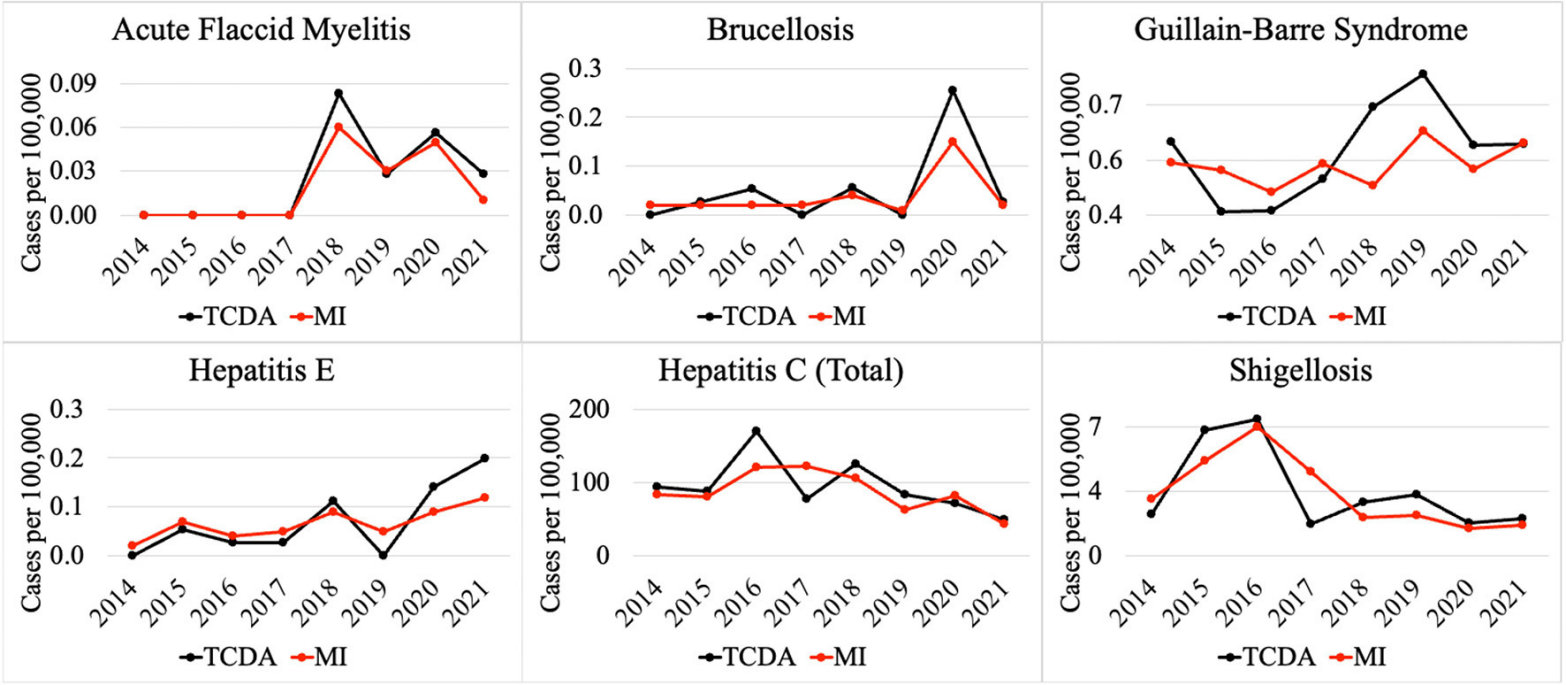
Comparison of selected CDs incidences (per 100,000) between TCDA and MI (ratio ≈ 1).

**Figure 6:**
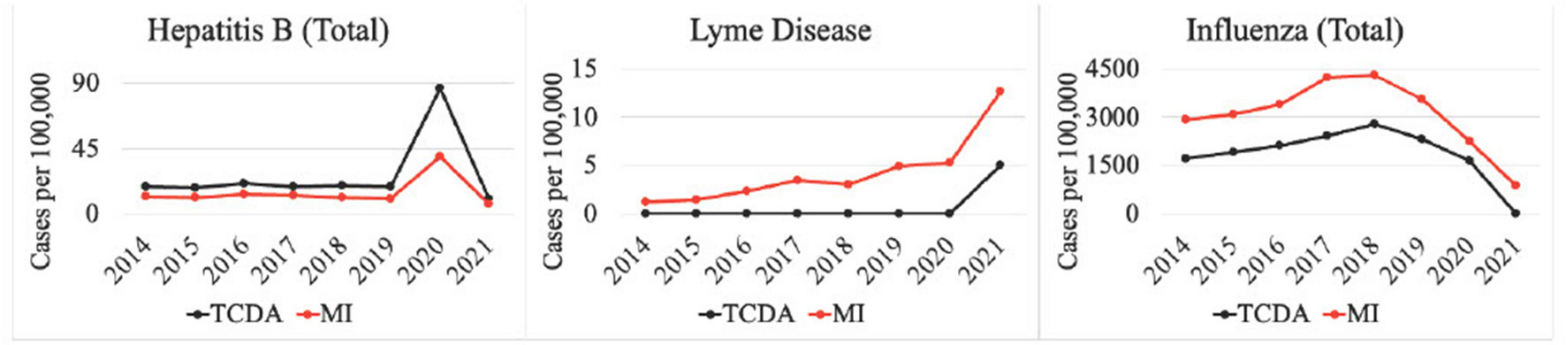
Selected CDs incidences potentially affected by the COVID-19 pandemic.

Weekly reports from the MDSS between 2014 and 2021 were accessed from the MDHHS website (michigan.gov/mdhhs). Data in the weekly reports were provisional, based on current data at the time that the report was published. Communicable disease incidence (per 100,000) for the state of Michigan are shown in Figure 2. Similar data was collected for the TCDA, including City of Detroit, and Wayne, Macomb, and Oakland Counties. Examples of disease trends between 2014 and 2017 are shown in Figures 3–**6**. MDSS weekly disease reports define the epidemiological “week” in concurrence with the CDC's Morbidity and Mortality Weekly Report (MMWR) (cdc.gov/mmwr), which runs from Sunday (day 1) to Saturday (day 7). All CDs were cross-referenced against multiple regulatory lists including the U.S. CDC's NNIDCL (cdc.gov/nndss), the U.S. EPA's CCL (epa.gov/ccl), and the U.S. CDC's bioterrorism agents list (cdc.gov/bioterrorism). Additionally, the detectability of the pathogens associated with each CD in human excreta and wastewater, which is crucial evidence for the applicability of wastewater surveillance for monitoring CDs, was investigated through an extensive literature review (Tables 1–4). R_0′_s were also collected through a literature review and are summarized in Table 5.

**Table 1:** MDHHS-reported conditions associated with viruses that can potentially be monitored with wastewater surveillance.

**Disease name**	**Virus potentially associated with the disease**	**Found in excrement**	**Found in wastewater**	**CDC NNIDCL?**	**EPA CCL?**
Acute flaccid myelitis (AFM)	West Nile, enteroviruses, other viruses	Yes	Yes	N	Y
Chickenpox (Varicella)	Varicella-Zoster Virus	Yes	Yes	Y	N
Chikungunya	Chikungunya Virus	Yes	N/A	Y	N
Coronavirus disease 2019 (COVID-19)	Severe acute respiratory syndrome coronavirus 2 (SARS-CoV-2)	Yes	Yes	Y	N
Dengue fever	Dengue Virus	Yes	Yes	Y	N
Flu like disease	Multiple viruses	N/A	Yes	N	N
Gastrointestinal illness	Multiple viruses, bacteria, parasites	Yes	Yes	Y	N
Hepatitis A	Hepatitis A Virus	N/A	Yes	Y	Y
Hepatitis B	Hepatitis B Virus	Yes	Yes	Y	N
Hepatitis C	Hepatitis C Virus	Yes	Yes	Y	N
Hepatitis E	Hepatitis E Virus	Yes	Yes	N	N
Human Immunodeficiency Virus (HIV) Infection	HIV virus	Yes	Yes	Y	N
Influenza	Influenza virus	N/A	Yes	Y	N
Measles	Measles virus	Yes	Yes	Y	N
Meningitis - Aseptic	Several kinds of viruses. Most Commonly nonpolio enteroviruses	N/A	Yes	N	N
Monkeypox	Monkeypox virus	Yes	Yes	N	N
Mumps	Mumps virus	Yes	N/A	Y	N
Norovirus	Norovirus	Yes	Yes	N	Y
Polio	Poliovirus	Yes	Yes	Y	N
Rubella	Rubella virus	N/A	Yes	Y	N
Shingles	Varicella-Zoster virus	Yes	Yes	N	N
VZ Infection, Unspecified	Varicella-Zoster virus	Yes	Yes	Y	N
West Nile Virus	West Nile virus	Yes	Yes	N	N
Yellow Fever	Yellow fever virus	Yes	N/A	Y	N
Zika	Zika virus	Yes	Yes	Y	N

MDHHS, Michigan Department of Health and Human Services; NNIDCL, National Notifiable Infectious Disease and Conditions List; CCL, Contaminant Candidate List. N/A indicates the information was unavailable at the time that the study was conducted. Data sources: (4–7, 13, 24, 32–55).

**Table 2:** MDHHS-reported conditions associated with bacteria that can potentially be monitored with wastewater surveillance.

**Disease name**	**Bacteria potentially associated with the disease**	**Found in excrement**	**Found in wastewater**	**CDC NNIDCL?**	**EPA CCL?**
Anthrax^*^	Bacillus anthracis	N/A	Yes	Y	N
Botulism^*^	Clostridium (botulinum, butyricum, baratii)	Yes	N/A	Y	N
Brucellosis^**^	Brucella spp.	N/A	Yes	Y	N
Campylobacter	Campylobacter spp.	Yes	Yes	Y	Y^∧^
Chlamydia (Genital)	Chlamydia trachomatis	Yes	N/A	Y	N
Cholera^**^	Vibrio cholerae	Yes	N/A	Y	N
CP-CRE	Enterobacter resistant to carbapenem	Yes	Yes	Y	Y
Gonorrhea	Neisseria gonorrhoeae	Yes	N/A	Y	N
Guillain-Barre Syndrome	Campylobacter jejuni, several viruses	N/A	Yes	N	Y
H. Influenzae Disease - Inv.	Haemophilus influenzae	Yes	N/A	Y	N
Legionellosis	Legionella pneumophila	Yes	Yes	Y	Y
Leprosy	Mycobacterium leprae	Yes	N/A	N	N
Leptospirosis	Leptospira spp.	Yes	N/A	Y	N
Listeriosis	Listeria monocytogenes	N/A	Yes	Y	N
Lymphogranuloma Venereum	Chlamydia trachomatis L1, L2, L3	Yes	N/A	Y	N
Nontuberculous Mycobacterium	Mycobacteria spp.	Yes	Yes	N	N
Paratyphoid Fever	Salmonella Paratyphii A, B, and C	Yes	Yes	N	N
Plague^*^	Yersinia pesits	N/A	Yes	Y	N
Psittacosis	Chlamydia psittaci	Yes	N/A	Y	N
Q Fever^**^	Coxiella burnetti	N/A	Yes	Y	N
Salmonellosis	Salmonella	Yes	Yes	Y	Y
Shiga Toxin-producing Escherichia Coli (STEC)	E. coli	N/A	Yes	Y	Y
Shigellosis	Shigella	Yes	Yes	Y	Y
Streptococcus Pneumoniae, Drug Resistant	Streptococcus pneumoniae	Yes	N/A	N	N
Streptococcus Pneumoniae, Inv	Streptococcus pneumoniae	Yes	N/A	N	N
Syphilis	Treponema pallidum	Yes	N/A	Y	N
Toxic Shock	Staphylococcus and streptococcus bacteria	N/A	Yes	Y	N
Trachoma	Chlamydia trachomatis	Yes	N/A	Y	N
Tuberculosis	Mycobacterium tuberculosis	Yes	Yes	Y	N
Typhoid Fever	Salmonella typhii	Yes	Yes	Y	N
VISA/VRSA	Staphylococcus aureus	N/A	Yes	Y	N

MDHHS, Michigan Department of Health and Human Services; NNIDCL, National Notifiable Infectious Disease and Conditions List; CCL, Contaminant Candidate List. N/A indicates the information was unavailable at the time that the study was conducted. ^*^indicates bioterrorism category A. ^**^indicates bioterrorism category B. Data sources: (56–86).

**Table 3:** MDHHS-reported conditions associated with parasites that can potentially be monitored with wastewater surveillance.

**Disease name**	**Parasite(s) potentially associated with the disease**	**Found in excrement**	**Found in wastewater**	**CDC NNIDCL?**	**EPA CCL?**
Amebiasis	Entamoeba histolytica	Yes	Yes	N	N
Cryptosporidiosis^**^	Cryptosporidium parvum	Yes	Yes	Y	N
Cyclosporiasis	Cyclospora cayetanensis	Yes	Yes	Y	N
Giardiasis	Giardia duodenalis	Yes	Yes	Y	N
Malaria	Plasmodium falciparum	Yes	N/A	Y	N

MDHHS, Michigan Department of Health and Human Services; NNIDCL, National Notifiable Infectious Disease and Conditions List; CCL, Contaminant Candidate List. N/A indicates the information was unavailable at the time that the study was conducted. ^**^indicates bioterrorism category B. The parasitic category is designated for pathogens caused by parasitic organisms, excluding fungal, bacterial, and viral pathogens. Data sources: (87–95).

**Table 4:** MDHHS-reported conditions associated with fungi that can potentially be monitored with wastewater surveillance.

**Disease name**	**Fungus potentially associated with the disease**	**Found in excrement**	**Found in wastewater**	**CDC NNIDCL?**	**EPA CCL?**
Blastomycosis	Blastomyces dermatitidis and gilchristii	Yes	N/A	N	N
Candida auris	Candida auris	Yes	Yes	Y	N
Cryptococcosis	Cryptococcus neoformans	N/A	Yes	N	N

MDHHS, Michigan Department of Health and Human Services; NNIDCL, National Notifiable Infectious Disease and Conditions List; CCL, Contaminant Candidate List. N/A indicates the information was unavailable at the time that the study was conducted. Data sources: (96–99).

**Table 5:** R_0_ values for 96 CDs.

**Disease name**	**R_o_ Value**	**Source**
Acute flaccid myelitis (AFM)	0	N/A
Amebiasis	7	(100)
Anthrax	1.251	(101)
Babesiosis++	1.56	(102)
Blastomycosis	0	N/A
Botulism (Total)	0	N/A
Brucellosis	0	N/A
Campylobacter	0	N/A
Candida auris	0	N/A
Chancroid	0	N/A
Chickenpox (Varicella)	11	(rcpi.ie)
Chikungunya	3.4	(103)
Chlamydia (Genital)	0.55	(104)
Cholera	2.15	(105)
Coccidioidomycosis	0	N/A
CP-CRE	0	N/A
Creutzfeldt-Jakob Disease	0	N/A
Cryptococcosis	0	N/A
Cryptosporidiosis	0	(106)
Cyclosporiasis	0	N/A
Dengue Fever	10	(107)
Diphtheria	7.2	(108)
Ehrlichiosis	0	N/A
Encephalitis	0	N/A
Flu Like Disease	1.5	(vdh.virginia.gov)
Gastrointestinal Illness	0	N/A
Giardiasis	4.181	(109)
Gonorrhea	0.89	(110)
Granuloma Inguinale	0	N/A
Guillain-Barre Syndrome	0	N/A
HIV	3.5	(netec.org)
H. influenzae Disease - Inv.	0	N/A
Hantavirus	0	N/A
Hemolytic Uremic Syndrome	0	N/A
Hemorrhagic Fever	1.62	(111)
Hepatitis A	0	N/A
Hepatitis B (Total)	9.175	(112)
Hepatitis C (Total)	2.12	(113)
Hepatitis E	6.5	(114)
Histoplasmosis	0	N/A
Influenza (Total)	1.5	(vdh.virginia.gov)
Kawasaki	0	N/A
Latent Tuberculosis	0	N/A
Legionellosis	0	N/A
Leprosy	2.75	(115)
Leptospirosis	1.52	(116)
Listeriosis (Total)	0	N/A
Lyme Disease	0	N/A
Lymphogranuloma venereum	3.5	(netec.org)
Malaria	0	N/A
Measles	15	(117)
Melioidosis	0	N/A
Meningitis - Aseptic	1.048	(118)
Meningitis - Bacterial Other	1.048	(118)
Meningococcal Disease	1.36	(119)
Monkeypox	2.1	(120)
Multisystem Inflammatory Syndrome	0	N/A
Mumps	11	(health.gov.au)
Nontuberculous Mycobacterium	9	(121)
Norovirus	2	(122)
Paratyphoid Fever	2.8	(123)
Pertussis	5.5	(124)
Plague	1.45	(125)
Polio	12	(126)
Psittacosis	0	N/A
Q Fever (Total)	0	N/A
Rabies	0	N/A
Reye Syndrome	0	N/A
Rheumatic Fever	0	N/A
Rickettsial Disease - Spotted Fever	1.7	(127)
Rickettsial Disease - Typhus	0	N/A
Rubella (Total)	6.6	(128)
Salmonellosis	0	N/A
SARS	2.11	(129)
STEC	1.5	(130)
Shigellosis	1.29	(131)
Shingles	0	N/A
Streptococcal Dis, Inv, Grp A	0	N/A
Streptococcal Toxic Shock	0	N/A
Streptococcus pneumoniae, Drug Resistant	1.5	(132)
Streptococcus pneumoniae, Inv	1.5	(132)
Syphilis (Total)	1.5	(133)
Tetanus	0	N/A
Toxic Shock	0	N/A
Trachoma	2.8	(134)
Trichinosis	0	N/A
Tuberculosis	8	(115)
Tularemia	1.57	(135)
Typhoid Fever	2.8	(123)
VISA	0	N/A
VRSA	0	N/A
VZ Infection, Unspecified	6.5	(136)
West Nile	0	N/A
Yellow Fever	0	N/A
Yersinia enteritis	0	N/A
Zika	3.8	(137)

N/A indicates the information was unavailable at the time that the study was conducted. Those pathogens for which the Ro is unknown will have zero (0) added to their cumulative score.

### 2.2. The CDWSRank system

The following sections demonstrate the design of the CDWSRank system and its associated parameters. The presence and absence of all CDs in regulatory lists including NNIDCL, WDR, and CCL, as well as being described as a bioterrorism agent, the association of the disease with a single or multiple pathogens, and detectability of pathogens in human wastewater were modeled as binary parameters. Quantitative parameters include: (1) clinical case trend in Michigan, (2) clinical case trend in the TCDA, (3) ratio of clinical case incidence between Michigan and the TCDA (geographic ratio), (4) annual clinical cases in Michigan, (5) annual clinical cases in the TCDA, and (6) the R_0_ (basic reproduction number) of the disease. The overall schematic of the parameters and weighting factors of each parameter is presented in Figure 1.

#### 2.2.1. Binary parameters

The presence or absence of CDs for each binary parameter was treated as a ×1 weighting factor (multiplier) and ×0 weighting factor (multiplier), respectively, which were then summed for the final ranking score. The CDC's NNIDCL provides comprehensive reporting of CDs that occur in the USA. Diseases that are reported in the NNIDCL are considered notifiable, but whether or not they are reported at the state level, varies (cdc.gov). Furthermore, internationally notifiable diseases reported in WHO's International Health Regulations (IHR), such as cholera, are also reportable in NNIDCL (cdc.gov). The IHR covers not only CDs but also other public health concerns including chemical and radiological threats (cdc.gov). All CDs were assessed for whether they are listed on the CDC's NNIDCL, and the corresponding presence or absence was marked with “Y” (presence in NNIDCL) or “N” (absence in NNIDCL). A multiplier of 1 was assigned to any CD's presence on NNIDCL. Similarly, the presence of a CD in the MDHHS Weekly Disease Report (WDR) was given a weighting factor or “multiplier” of 1.

The EPA's CCL includes drinking water contaminants that are recognized or expected to occur in public water systems and are not currently subject to EPA drinking water regulations (epa.gov). The EPA uses the CCL to identify priority contaminants for regulatory decision-making and information gathering (epa.gov). The EPA announced Draft CCL 5 on July 19, 2021, followed by the publication of Final CCL 5 on November 14, 2022 (epa.gov). All CDs were assessed for whether they appear on EPA CCL 5, and the corresponding presence or absence was marked with a “Y” (presence in CCL) or “N” (absence in CCL).

The CDC classifies bioterrorism agents into 3 categories, namely, A, B, and C, depending, primarily, on how easily the diseases can be transmitted and the severity of illness (cdc.gov). Agents in category A are considered of the highest risk, as they can be easily transmitted within human populations and can result in high death rates and significant public health impacts. Examples include anthrax and plague. Agents in category B have the second highest priority risk, as they are moderately easy to spread and can result in moderate morbidity rates. Examples include Q fever and typhus fever. Agents in category C are considered the third highest priority risk and they can easily spread among humans and cause health impacts (cdc.gov). Examples include hantavirus and Nipah virus. The presence of CDs as CDC-defined bioterrorism agents was marked with “^*^” for category A and “^**^” for category B. A weighting factor or multiplier of 1 was assigned to a CD listed as a CDC bioterrorism agent, regardless of category.

The detectability of pathogens causative of CDs in human wastewater is crucial to the successful implementation of wastewater surveillance. Following extensive literature reviews, the detectability of the causative pathogen in excreta or wastewater was marked with a “Y” (detectable), “N” (non-detectable), or “N/A” (data unavailable) in Tables 1–4. For the final ranking score, a multiplier of 1 or 0 was given to CDs with a causative pathogen that is detectable or non-detectable, respectively, in excreta and/or wastewater.

The binary parameter of disease associated with single or multiple pathogens considers the exact source of a causative pathogen of a CD. In this system, CDs with multiple causative pathogens would make them nearly impossible to be determined or detected. Therefore, CDs with multiple causative pathogens were assigned a multiplier of 0 at the final ranking score, to moderate the over-ranking of these CDs. A final ranking multiplier of 1 was assigned to CDs with a single causative pathogen.

#### 2.2.2. Quantitative parameters

Quantitative parameters include: (1) clinical case trend in Michigan, (2) clinical case trend in the TCDA, (3) ratio of clinical case incidence between Michigan and the TCDA (geographic ratio), (4) annual clinical cases in Michigan, (5) annual clinical cases in the TCDA, and (6) the R_0_ (basic reproduction number) of the disease. Clinical case trends in Michigan as a whole and in the TCDA specifically, were determined by calculating the correlation R-value between disease incidence (per 100,000) each year (2014 to 2021) and the given year, for all CDs. The weighting factor or multiplier of 1.5 and 2.5 were assigned to clinical case trends in Michigan and the TCDA, respectively, providing greater emphasis on the TCDA.

The ratio of clinical case incidence between Michigan and the TCDA is assessed through calculating case incidence (per 100,000) for each CD, for the state of Michigan, then the TCDA. Next, the ratio of these values is calculated as the quotient of Michigan cases and TCDA cases, done for each year in the study period. Finally, the average of annual ratios was calculated, and each CD was assigned a value of 1 if the average was less than 1 (indicating that the CD was more prevalent in the TCDA than the state of Michigan as a whole). A CD was assigned a value of 0 if the ratio was equal to, or greater than 1. A weighting factor or “multiplier” of 2 was given to this metric.

Clinical cases in Michigan and in the TCDA were determined by computing the decadic log of the average clinical caseload for the years studied. Taking the common logarithm was necessary as clinical caseloads varied greatly in magnitude; this operation, therefore, allowed for the comparison of CDs even with disparate magnitudes of caseloads, while still preserving accurate variation measures. The weighting factor or “multiplier” of 1.5 and 2.5 were assigned to clinical cases in Michigan and the TCDA, respectively, providing greater emphasis on the TCDA.

The R_0_ of CDs were determined through literature investigation (Table 5). This parameter was included to increase the ranking score of CDs that can be transmitted efficiently, through person-to-person contact (167). This parameter prioritizes CDs that have the potential to spread rapidly. This parameter was given a weighting factor of 1.

#### 2.2.3. Overall CDWSRank system ranking score

An overall ranking score (R_CD_) of the CDWSRank system for CDs is calculated using the following Eq. (1), where *R*_*CD*_ is the overall ranking score of the *i*^th^ CD, *W*_*i*_ is the weighting factor for binary parameters, *N*_*i*_ is the weighting factor for quantitative parameters, *B*_*i*_ represents binary parameters, *Q*_*i*_ represents quantitative parameters, *D*_*i*_ represents the detectability of causative pathogens in human excreta or wastewater, and *M*_*i*_ represents the association of a CD with a single or multiple pathogens.


(1) $$R_{\text{CD}}=(W_{i}\sum_{i=1}^{n}B_{i}+N_{i}\sum_{i=1}^{m}Q_{i})\times D_{i}\times M_{i}$$

An equation for calculating an overall rank score of the *i*^th^ CD with all binary and quantitative parameters displayed, can be expressed as follows:

R_CD_ = [1 × (NNIDCL) + 1 × (WDR) + 1 × (CCL) + 1 × (Bioterrorism) + 2 × (Geographic ratio) + 1.5 × (Clinical case trend in Michigan) + 2.5 × (Clinical case trend in the TCDA) + 1.5 × (Clinical case in Michigan) + 2.5 × (Clinical case in TCDA) + 1 × (R_0_)] × [1 × (Detectability in human excreta or wastewater)] × [1 × (Association of disease with single or multiple pathogens)] (2)

For example, an overall rank score of SARS-CoV-2 can be computed as: [1 × (1) + 1 × (1) + 1 × (0) + 1 × (0) + 2 × (1) + 1.5 × (0.57) + 2.5 × (0.6) + 1.5 × (5.39) + 2.5 × (4.98) + 1 × (2.11)] × (1) × (1) = 29.

### 2.3. Wastewater surveillance concentration methods based on pathogen type

In addition to the development of the CDWSRank system, a comprehensive literature review was conducted to summarize appropriate wastewater sample concentration surveillance methods based pathogen type, namely: bacterial, fungal, parasitic, and viral (Table 6).

**Table 6:** Concentration methods for wastewater surveillance by pathogen type.

**Pathogen type**	**Concentration methods**	**Reference**
Bacterial	Centrifugation	(138–141)
	Membrane filtration	(142, 143)
	Precipitation and filtration	(144)
Fungal	Centrifugation and culture	(145, 146)
	Plate culture growth	(147–149)
Parasitic	Centrifugation	(150–156)
	Filtration and centrifugation	(157, 158)
Viral	Aluminum-driven flocculation	(159)
	Concentrator instrument	(2)
	Centrifugation	(160, 161)
	Electronegative membrane vortex	(162)
	Filtration	(160, 163)
	Membrane adsorption	(159, 164)
	Organic flocculation	(163)
	PEG	(5, 160, 162, 163)
	Precipitation	(164)
	Ultracentrifugation	(164)
	Ultrafiltration	(8, 11, 160–162, 164, 165)
	VIRADEL	(4–8, 17, 165, 166)
	Without pre-treatment/concentration	(16)

## 3. Results

### 3.1. Classification of CDs

Tables 1–3 present viruses, bacteria, parasites and fungi that are detectable in human excrement or wastewater, indicating their potential to be monitored by wastewater surveillance. Notably, some of the listed pathogens were successfully detected in worldwide wastewater samples, with disease incidence monitored using wastewater surveillance. These include dengue virus (32), hepatitis B (33), monkeypox virus (22–24), norovirus (168, 169), Poliovirus (19, 20), SARS-CoV-2 (2, 4–7, 10, 16), yellow fever virus, and zika virus (32).

Twenty-five CDs are associated with viral pathogens, including chickenpox, COVID-19, monkeypox, norovirus, West Nile fever and so on (Table 1). The viruses that are associated with the diseases are also summarized in Table 1. For instance, varicella-zoster virus is the causative agent of chickenpox. Notably, only 3 of the 25 viruses, including acute flaccid myelitis-related enterovirus, hepatitis A, and norovirus, appear on the EPA's CCL. Some viral diseases can be found on the CDC's NNIDCL, including COVID-19, HIV/AIDS, and Zika. No viral CDs in the list are classified as CDC bioterrorism agents. Table 2 shows 31 CDs associated with bacterial pathogens, including anthrax, cholera, gonorrhea, plague, syphilis, and so forth. The bacteria that are potentially associated with the diseases were also summarized in Table 2. For instance, clostridium (botulinum, butyricum, baratii) is the potential causative agent associated with botulism. Seven of the 31 bacteria are listed on the EPA's CCL, including chlamydia, CP-CRE, Guillain-Barre syndrome, legionellosis, salmonellosis, STEC, and shigellosis. And 25 of the 31 of the bacterial-related CDs are listed on the CDC's NNIDCL. Six of 31 bacterial-related CDs are not listed on the CDC's NNIDCL, including Guillain-Barre syndrome, leprosy, non-tuberculous mycobacterium, paratyphoid fever, and streptococcus pneumoniae. Among all bacterial CDs, anthrax, botulism, and plague are listed in bioterrorism category A, while brucellosis, cholera, and Q fever are listed in bioterrorism category B. Table 3 includes 5 parasitic CDs that can be detected in either human excreta or wastewater. The potentially causative agents of these diseases were also summarized in Table 3. For instance, cryptosporidium parvum is the parasite associated with cryptosporidiosis. None of pathogens related to parasitic CDs are listed on the EPA's CCL, and 4 of them are listed on the CDC's NNIDCL, including cryptosporidiosis, cyclosporiasis, giardiasis, and malaria, expect for amebiasis. Cryptosporidiosis is listed in the CDC's bioterrorism category B. Lastly, Table 4 shows 3 fungal-related CDs, including blastomycosis, cryptococcosis, and candidiasis. The fungi associated with the diseases are summarized in Table 4. For instance, blastomyces dermatitidis and gilchristii are the potential causes of blastomycosis. None of them are listed on the EPA's CCL and only candidiasis (candida auris) was listed on the CDC's NNIDCL (Table 4).

### 3.2. Observations of CDs' incidence and trend

#### 3.2.1. Comparison of CD incidence in the TCDA vs. the state of Michigan

All CD incidences (per 100,000) from 2014 to 2021 in Michigan are demonstrated in Figure 2. Influenza, “influenza-like” or “flu-like” diseases, chlamydia, gonorrhea, and gastrointestinal illness (GI) have among the highest average incidences in Michigan.

Notably, multiple CDs presented lower incidences (per 100,000) in the TCDA than in broader Michigan (Figure 3). GI presented much higher cases per 100,000 in Michigan than in TCDA. Between 2017 and 2019, more than 1,400 incidences per 100,000 were observed in Michigan. In contrast, during the same period, approximate 400 incidences per 100,000 were observed in TCDA (Figure 3). Likewise, incidences per 100,000 of cryptosporidiosis, giardiasis, and norovirus were observed as much as twice higher in Michigan than in TCDA.

On the contrary, multiple CDs presented higher incidences (per 100,000) in the TCDA than in broader Michigan (Figure 4). CDs, such as gonorrhea, which can cause severe and permanent health issues (cdc.gov), has increased continuously and dramatically from 5,245 cases in 2014 to 12,034 cases in 2020 (and slightly decreased to 10,483 cases in 2021) in the TCDA. Gonorrhea incidence in TCDA is approximately five times higher than the rest of Michigan (Michigan.gov). Likewise, sextually transmitted diseases such as HIV, syphilis, and chlamydia were observed with consistent higher incidences per 100,000 in TCDA than in statewide Michigan (Figure 4). Also, West Nile fever incidences per 100,000 have increased dramatically in TCDA from 2019 to 2020 (Figure 4).

Figure 5 demonstrates selected CDs with approximately the same disease incidence (per 100,000), between the TCDA and Michigan, including AFM, brucellosis, Guillain-Barre syndrome, hepatitis E and C, as well as shigellosis.

#### 3.2.2. Potential impact of COVID-19 pandemic on CDs

Multiple CDs were potentially affected by the COVID-19 pandemic (Figure 6). For instance, cases of hepatitis B surged from 675 (Michigan) and 1,081 (TCDA) in 2019, to 3,064 (Michigan) and 4,007 (TCDA) in 2020, during the inchoate stages of the COVID-19 pandemic. Afterwards, incidences in both Michigan and the TCDA decreased significantly, during COVID-19 stabilization, suggesting that a pandemic could cause an impact on disease incidence. The pandemic also affected the incidence of several vector-borne diseases, for example Lyme disease. Lyme disease surged in both Michigan as a whole and the TCDA between 2020 and 2021 (Figure 6). The incidence of influenza per 100,000 individuals in both TCDA and Michigan has been consistently decreasing since 2018. However, the decrease has been particularly significant from 2020 to 2021, concurring with the global spread of COVID-19. This may suggest that the health control measures implemented in response to the COVID-19 pandemic, such as shelter-in-place orders and social distancing, have had a positive impact on reducing the incidence of influenza.

### 3.3. Overall ranking

**Figure 7:**
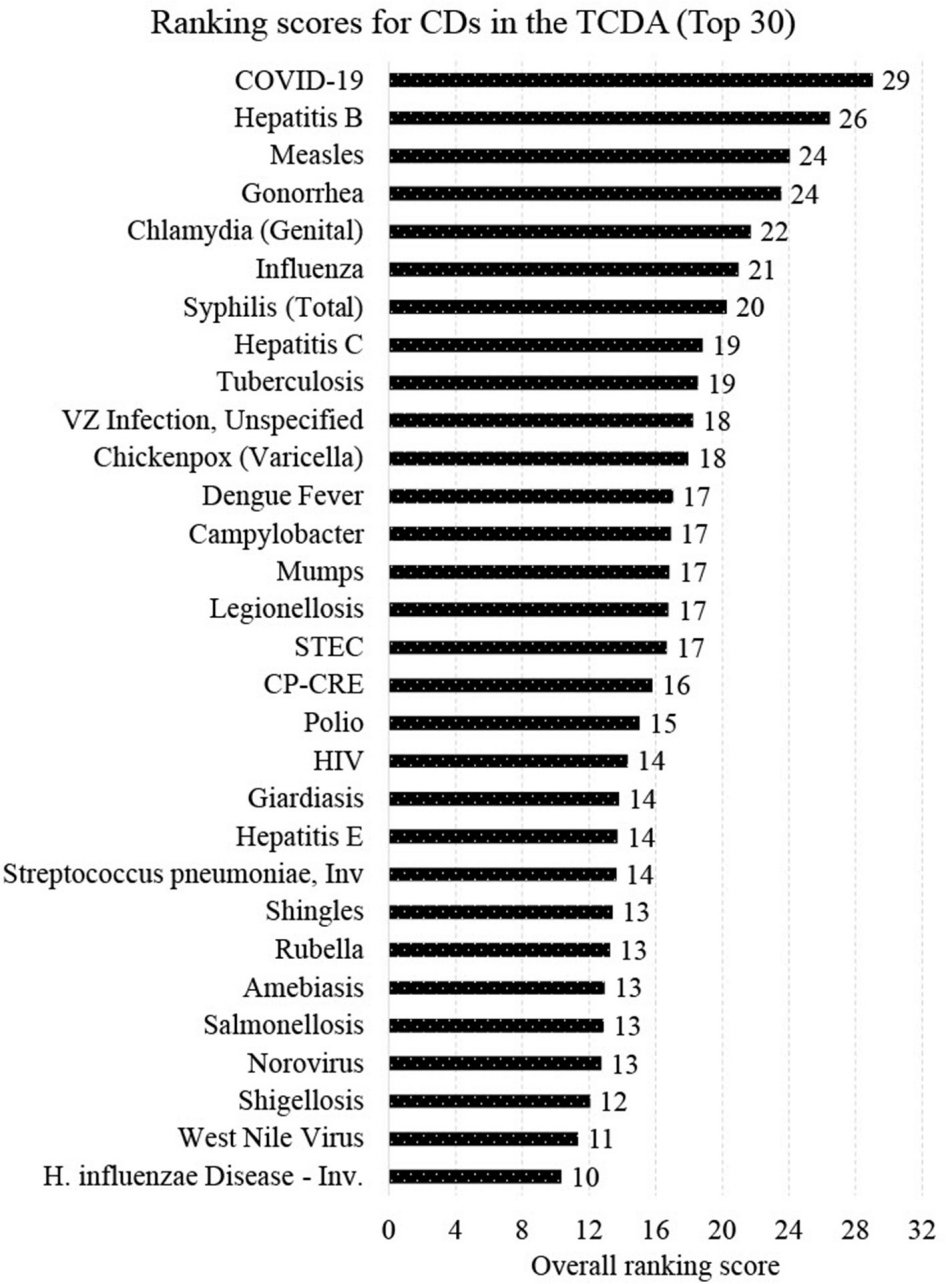
Top 30 CDs by CDWSRank system for prospective wastewater surveillance in the TCDA.

Figure 7 presents the final ranking (top 30 out of 96 CDs) generated from the CDWSRank system, prioritizing wastewater surveillance target applications in the TCDA. Several CDs caused by viruses that are detectable in human excreta or wastewater were among the top 30 listed. These include COVID-19 (ranked 1^st^), hepatitis B (ranked 2^nd^), measles (ranked 3^rd^), influenza (ranked 6^th^), hepatitis C (ranked 8^th^), polio (ranked 18^th^), HIV/AIDS (ranked 19^st^), hepatitis E (ranked 21^st^), and norovirus (ranked 27^th^). Among the top 30 ranked CDs, some did not present relatively high incidences but were prioritized upon using the CDWSRank system. Examples include measles, polio, HIV/AIDS, hepatitis E, and norovirus, suggesting that such CDs require significant attention by wastewater surveillance practitioners, despite their relatively low incidence rates in the geographic study area in recent years.

Though not unexpected, the highest ranked CDs are those that do not spread solely by direct contact with animals, but rather those that are transmitted from person to person or from food or fomites. Only one vector-borne disease appears within the top 30, which is West Nile fever (ranked 29^th^). Over 50% (16/30) of CDs in the top 30 are either foodborne or STIs.

It is worth noting that 4 of the top 30 ranked CDs are known to health agencies to be vaccine preventable, highlighting the need for surveillance to warn against conditions that are not easily preventable, or those that could be particularly devastating to those not able to be immunized, such as infants or the immunodeficient. One CD ranked by this system was assigned a negative R_CD_, melioidosis. This indicates that, though detectable using wastewater surveillance methods, this disease has been trending downward in the geographic areas and timeframe of this study, precluding it as a priority for monitoring.

Additionally, certain CDs (mentioned in Sections 3.2.1 and 3.2.2) received a ranking score of 0 since a multiplier of 0 for binary parameters was assigned. Lyme disease, for example, received a score of 0 since the detectability of Lyme disease in excreta or wastewater was set to 0. It was set to 0 because at the time of this study there were no published reports available indicating the ability to detect the bacteria (*Borrelia burgdorferi* and *Borrelia mayonii*) that causes Lyme disease in excreta or wastewater. As research efforts of the scientific community progress this may change.

## 4. Discussion

### 4.1. Differences of CDs in TCDA and state of Michigan

Differences in incidence among CDs in the TCDA vs. the state of Michigan demonstrate epidemiological trends that differ, possibly due to population density, wildlife/ecology, climate, socioeconomic and racial inequities, cultural or behavioral differences, age distribution, and access to healthcare and/or medical insurance (170–174). The ranking system results focus on TCDA which is an urban area with high-density population. However, as of 2021 (175), approximately 1.8 million residents, which accounts for nearly 20 percent of Michigan's population, live in rural areas. Consequently, Michiganders as a whole face a relatively elevated risk of contracting CDs such as cryptosporidiosis, giardiasis, and norovirus (Figure 3).

Residents in rural areas may have limited accessibility to medical care for diseases that require extensive or sophisticated care regimens (176). A study demonstrated possible causes for disparities between urban and rural areas by comparing outdoor time, where longer outdoor time were spent by rural residents than their urban counterparts (174), potentially creating an elevated risk of being infected by zoonotic pathogens. In rural areas, zoonotic diseases are of particular concern for farm workers, especially those working with livestock (177). In addition to zoonotic disease, residents of rural areas of Michigan are of great concern for vector-borne diseases, such as babesiosis (Figure 3), and others (178). It is important to note that human behavior, such as water related human activities, can also impact the transmission of vector-borne diseases, in addition to the effects of a warming climate in Michigan, especially the TCDA area (179). For example, higher average incidence of West Nile fever in the TCDA than in statewide Michigan can be attributed to both factors (180, 181).

Multiple CDs presented higher incidences per 100,000 in TCDA than in statewide Michigan, such as HIV and syphilis. This could possibly be related to a limited access to healthcare among the socioeconomically disadvantaged and racial minorities in TCDA (182). There are multiple causes of higher disease incidence of HIV and other STIs in TCDA, such as gonorrhea and syphilis (Figure 4). Briefly, a recent investigation indicated that elevated HIV prevalence in the TCDA was associated with minorities, gay and bisexual populations up to 29 years old, and the socioeconomically disadvantaged, such as those experiencing homelessness, poverty, and unemployment (170). It is worth noting that this trend is observed nationwide (183, 184). Researchers have also found that TCDA had a TB incidence twice than that of Michigan, affected by both racial inequity and places of interaction (185).

### 4.2. Impact of COVID-19 pandemic on CDs in TCDA and state of Michigan

Incidences per 100,000 of diseases such as hepatitis B, influenza and others, in both Michigan and the TCDA changed significantly, during COVID-19 inception, suggesting that a pandemic could cause an impact on disease incidence (Figure 7). This was corroborated in recent studies (186–188), and has been shown in countless epidemics worldwide (189, 190). Interestingly, several CDs whose incidences fell during the pandemic were those that traditionally rose in the other reported years, such as influenza. It is likely that reduced human contact and heightened hygiene in response to COVID-19 may have caused the dramatic decrease (191). On the contrary, Lyme disease surged in both Michigan as a whole and the TCDA between 2020 and 2021 (Figure 6). This may be attributable to an increasing number of outdoor recreational activities as result of diminished indoor options, due to COVID-19 social distancing restrictions (192). Another potential explanation for the pandemic's effect on CD incidence is that some CDs are caused by opportunistic pathogens that reactivate in a host when an individual's immune response is weakened, often by another pathogenic condition (193). The renewed prevalence of these CDs can be a direct effect of COVID-19 disease, creating the conditions of pathogen reactivation or new infections (194).

Studies have investigated the impact of the COVID-19 pandemic on sexually transmitted infections (STIs), such as syphilis (Figure 4) (195, 196). The disease incidence (per 100,000) of syphilis increased significantly between 2020 and 2021 in both the TCDA and broader Michigan, amid the pandemic (Figure 4). Potential causes may include the diversion of funding and health resources from STI programs, shutdown of STI clinics, less available treating physicians, a reticence to appear in-office to meet clinicians, and longer laboratory turnaround times (195). It is worth noting that during the COVID-19 pandemic, many health reporting systems faced challenges due to the increased workload and limited resources in the public health workforce (197). This may have led to delays in reporting some diseases or with lower-quality data. However, it is important to note that COVID-19 has also resulted in improvements in health reporting systems in some areas, as public health agencies and governments have recognized the importance of timely and accurate reporting of disease data (198). The impact on health reporting systems by COVID-19 pandemic varied depending on the region, the disease, and the public health response to the pandemic.

### 4.3. Wastewater surveillance for viral CDs

CDWSRank placed 16 viral CDs in the top 30 for wastewater surveillance (Figure 7). Hepatitis B, for example, ranked 2^nd^ (Figure 7). Recently, researchers conducted wastewater surveillance to monitor hepatitis B in 19 cities across China, after clinical cases had increased dramatically (33). The wastewater surveillance results were consistent with the prevalence reported in surveys, indicating that estimating Hepatitis B prevalence through wastewater surveillance is feasible in large cities in Southern China. Hepatitis C ranked 8^th^ in CDWSRank for the TCDA region. Its RNA was detected and quantified in human fecal specimens in multiple studies, suggesting a significant potential for using wastewater monitoring as a tool for detecting hepatitis C virus (199). Chickenpox (ranked 11^th^) has been persistent in the statewide Michigan between 2014 and 2021, as shown in Figure 2. A few studies have attempted to test human bodily fluids, particularly urine, for monitoring varicella-zoster virus (which causes chickenpox and shingles), and other similar pathogens, such as in the Poxviridae family (34, 200). Notably, belonging to the same orthopoxvirus genus as varicella-zoster (201), the monkeypox virus has been spreading worldwide (outside of its traditional range) since May 2022. The virus has been detected in wastewater in Rome, Italy (23), and California, USA (24), showcasing the immense potential of wastewater surveillance as a tool for monitoring viruses in the Poxviridae family (200).

Viral pathogens, such as measles virus (measles is ranked 3^rd^) and varicella-zoster (shingles is ranked 23^rd^) were detected in urine specimens, indicating their potential to be monitored through wastewater surveillance as well (35, 202). Influenza, which ranked 6^th^ on CDWSRank, was investigated in previous studies regarding the potential of wastewater surveillance (203).

Notably, polio ranks 18^th^ in our CDWSRank system primarily due to its high R_0_ value, indicating that it has the potential to spread widely and quickly. Although polio cases have not been identified in Michigan between 2014–2021, the disease can have severe health consequences and can be dangerous if it emerges. It is worth noting that the data published by the MDHHS is subject to yearly review. New information and inclusion of recent data could potentially affect the ranking of polio or any other CDs in our CDWSRank system. Polio's inclusion in our system is based on its potential to pose a significant public health threat, highlighting the importance of ongoing disease surveillance efforts to prevent the resurgence of CDs like polio. Overall, our CDWSRank system is designed to indicate which diseases should be prioritized in the context of wastewater surveillance for TCDA based on local clinical data and other parameters such as R_0_.

### 4.4. Wastewater surveillance for bacterial, fungal, and parasitic CDs

CDWSRank placed 12 bacterial CDs ranked in the top 30 (Figure 7). These include tuberculosis (ranked 9^th^), CP-CRE (ranked 17^th^), legionellosis (ranked 15^th^), salmonellosis (ranked 26^th^), and shigellosis (ranked 28^th^), all detecteble both in human excreta and wastewater. Also, campylobacter (ranked 13^th^) was identified as a highly-sensitive pathogen for wastewater surveillance (204). Bacterial pathogens, such as *Chlamydia trachomatis* can be detected in wastewater (205). Despite being detectable in human excreta and wastewater, paratyphoid fever, Q fever, and typhoid fever were not ranked among the top 30 CDs.

Only two parasitic CDs, giardiasis (ranked 20^th^) and amebiasis (ranked 25^th^) ranked among the top 30. No fungal CDs were ranked among the top 30 CDs. Despite this, fungal CDs, including blastomycosis and cryptococcosis have great potential to be monitored using wastewater, as they can be detected in either human excreta or wastewater (Table 4).

### 4.5. Strengths and limitations of CDWSRank system

The goal of this study is to develop a quantitative prioritization system for wastewater surveillance of CDs in the TCDA. Several studies have developed methodologies to rank the threat of CDs with different scopes and methodologies (28–31). However, these studies have many limitations in their ranking systems which were refined and improved by the CDWSRank system.

Firstly, these ranking systems did not include parameters such as actual disease cases and basic reproduction numbers (R_0_) for CDs (28–31). For instance, Balabanova et al., applied criteria such as incidence rate to prioritize 127 CDs in Germany (28). However, the study did not include the actual annual incidence number of the CDs. Instead, the importance of incidence for each disease was evaluated by weights given by experts. In our CDWSRank system, the actual disease incidence data between 2014 and 2021 for 96 CDs were extensively investigated and included in the system. Besides, in this study we investigated the R_0_ for 96 CDs and incorporated them in the system when available.

Secondly, existing ranking systems relied heavily on experts' opinions on weighting the parameters when ranking the diseases (28–31). For instance, Cardoen et al., proposed a ranking system for 51 zoonotic agents which replied on scores given by 35 scientific experts in the field of animal and public health, food, clinical microbiology, and epidemiology (30). Likewise, Humblet et al., applied multicriteria decision-making methodologies based on expert opinions and data to rank 100 infectious diseases, in a system that included 57 criteria and 5 categories encompassing epidemiology, economy, public health, society, and prevention/control (31). The systems are affected by individual opinions of experts evaluating qualitative parameters. Experts' opinions could be subject to bias, which can affect the final ranking results. The subjective nature of weighting parameters by individuals for some criteria, such as public health impact, animal health impact, and food impact, can lead to uncertainty and variation in final ranking scores depending on individual interpretations of these parameters (30). In contrast, to circumvent the bias of subjective opinions of experts, we designed the CDWSRank system based on a data-driven approach that considers critical factors including quantitative parameters of disease incidence and trend, geographical ratio, and R_0_ for all CDs. In this way, the proposed ranking system differs from existing systems that are primarily based on the subjective, albeit expert, opinions. Besides, the weights given by experts for the specific locations can be hardly applied to other areas. However, by replacing the quantitative parameters in CDWSRank system, it can be applied beyond TCDA to other locations with accessible data. For instance, the clinical case trend in the State of Michigan and TCDA can be replaced by clinical disease databases based on different geographical information, henceforth enhancing the CDWSRank system's potential for wider applications.

Thirdly, the ranking systems in previous studies were designed for specific events or areas, which can be hardly applied beyond their scope. For instance, Balabanova et al. (28) included notifiable diseases in Germany and reportable diseases within the European Union. Likewise, Economopoulou et al. (29) focused only on the risk of CDs associated with the hosting of the London 2012 Olympic Games. To circumvent those biases, the 96 CDs included in CDWSRank system were selected based on U.S. CDC reportable disease lists and other governmental lists including the EPA CCL and CDC Bio-terrorism List, and local disease report lists including MDHHS WDR, which distinguishes it from all previous ranking systems for ranking CDs (28–31). This proposed ranking system is highly adaptable to other regions, especially those with similar reporting models which most states in the United Sates have, as a result of the CDC National Notifiable Disease Surveillance System requirements. Furthermore, to the best of our knowledge, there have been no published studies ranking CDs of public health importance that can be monitored using wastewater surveillance.

The goal of this study was to develop a prioritization system for wastewater surveillance of CDs in the TCDA. Limitations of this study are expounded below. Firstly, a multiplier of 0 was applied to a given CD if their causative pathogen has not been detected in wastewater or human excreta according to published studies thus far. This excludes potentially harmful CDs which can result in severe public health consequences, such as anthrax, hantavirus, and plague. Secondly, the weighting factors or multipliers for both binary and quantitative parameters were determined by researchers of this study and specifically designed with an emphasis on the TCDA. Nonetheless, weighting factors are adjustable and can vary across studies and regions with dissimilar research emphases. Thirdly, data unavailability limited the parameter types that could be involved in the proposed ranking system. For instance, mortality rate, case fatality, or incidence rate of some CDs could not be located in any published studies or publicly-available datasets for the TCDA. Additionally, due to a lack of R_0_ information on some CDs, the ranking system may have disregarded diseases that are potentially harmful to human health but that do not yet have an established, specific R_0_. R_0_ values are situation-dependent and can significantly affect the rank (167). Besides, the CDWSRank system is limited since it does not explore the connection between severity and economic impact of the diseases ranked in this study. The severity of the disease in many instances would vary significantly with access to health care and the economic impact would vary with the severity. Despite the researchers' initial attempts to include parameters of mortality rate and severity, very few studies were found that adequately quantified these values in the TCDA region. It is, however, possible to include these parameters when adapting the CDWSRank system for a different locale if those data are available in the new area studied. Another significant limitation on the CDWSRank system is its reliance on case data being publicly and readily available. The implications of this limitation become particularly salient in locations where clinical data and information for reportable diseases are unavailable. However, as the CDWSRank system did produce a ranking score for Monkeypox, a disease without the case numbers published at the time of study, it is evident that the system can still create a ranking based on the other parameters. Hence, the CDWSRank system retains its utility in settings where access to data is restricted.

Social determinants of health such as socioeconomic status, environment, race and ethnicity, gender, culture, and access to health care would be other parameters for future development of the CDWSRank system. However, measuring and quantifying these factors for all 96 CDs in TCDA pose significant challenges, given the limited availability and accessibility of relevant data. Nonetheless, the insights generated by the CDWSRank system can be particularly valuable for guiding wastewater surveillance of emerging CDs which is beneficial for socioeconomically disadvantaged communities with limited healthcare access or traditional surveillance systems. Nevertheless, it is critical to note that as the aforementioned constraints become known, updating the CDWSRank system becomes necessary.

It is worth noting that some of the diseases of concern are seasonal (such as influenza) or rare (such as polio) and therefore only occasional surveillance may be recommended. In addition, some CDs, such as chlamydia, gonorrhea, and HIV, prioritized by CDWSRank system in TCDA are associated not only with urban areas, but also with socioeconomical and racial inequality, which can skew statistical designs. Social determinants of health, such as poverty, poor housing conditions, lack of access to healthcare, can disproportionately affect certain racial or ethnic groups and increase their risk of contracting and transmitting communicable diseases (170–173). For example, individuals living in crowded and unsanitary conditions are more likely to contract infectious diseases like TB or hepatitis A (206, 207). Therefore, surveillance of specific regions of concern may be recommended.

### 4.6. Future directions

In the State of Michigan, as in multiple other regions across the nation, the COVID-19 pandemic prompted the creation of wastewater surveillance networks. As the primary health focus shifts away from COVID-19 these currently available networks and their infrastructure and resources can be adapted to monitor other emerging diseases. This study offers a tool for transitioning to wastewater surveillance programs beyond COVID-19. By identifying and ranking the CDs that pose the most significant risk to public health in TCDA, the CDWSRank system provides a methodological tool and critical information that can help public health officials and policymakers allocate resources more effectively. This information can be used to prioritize disease surveillance efforts and ensure that public health interventions are targeted at the most potentially urgent threats.

Furthermore, with regards to the extension of the CDWSRank system's applicability beyond the TCDA region, it is worth noting that the quantitative parameters heavily rely on local clinical data, while the binary parameters are primarily developed from regulatory lists obtained from local health departments as well as from U.S. governmental agencies. It is worth mentioning that all states in the U.S. are mandated to report to the C.D.C. and have their respective local health departments responsible for reporting notifiable diseases. Therefore, extending the application of the CDWSRank system to other regions within the U.S. would be relatively straightforward.

## 5. Conclusion

In this study, we developed a comprehensive and effective ranking system (CDWSRank) of wastewater surveillance prioritization for 96 CDs in the Tri-County Detroit Area (TCDA), Michigan, USA. The CDWSRank system comprises 6 binary and 6 quantitative parameters, with CDs classified into four groups: viral, bacterial, fungal, and parasitic. Critical regulatory lists, including the CDC's NNIDCL, MDHHS's WDR, EPA's CCL, and CDC's bioterrorism agents list were incorporated into the CDWSRank system. Disease incidences and trends of reportable CDs in the TCDA and broader state of Michigan were also incorporated into the system. Disparities in incidences of CDs were identified between the TCDA and state of Michigan, indicating epidemiological differences. Appropriate sampling and sample concentration methods for wastewater surveillance application were summarized as per our four categories, viral, bacterial, fungal, and parasitic.

The CDWSRank system is one of the first of its kind with the potential to prioritize resources and efforts toward monitoring and preventing the spread of CDs through wastewater surveillance. It helps researchers and public health practitioners to identify CDs that at a higher risk of disease transmission and prioritize monitoring efforts to mitigate their spread. The CDWSRank system provides an evidence- and data-based approach to decision making, ensuring the resources are allocated for wastewater surveillance beyond the COVID-19 pandemic. Ultimately, the development and implementation of the CDWSRank system for CDs can help reduce the impact of CDs on public health and promote broader applications of wastewater surveillance for public health benefits. CDWSRank can and should be adopted for ranking CDs in other geographical locations, with updated etiological and epidemiological information.

## Data availability statement

The original contributions presented in the study are included in the article/supplementary material, further inquiries can be directed to the corresponding author.

## Author contributions

ZG and LZ: methodology, investigation, data acquisition, data curation, formal analysis, visualization, writing—original draft, writing—review and editing, and are co-led manuscript development. RF and RD: writing—review and editing. JN: funding acquisition and writing—review and editing. IX: conceptualization, funding acquisition, methodology, investigation, project administration, resources, supervision, and writing—review and editing. All authors contributed to the article and approved the submitted version.
